# New Appliances and Things Medical

**Published:** 1905-06-17

**Authors:** 


### NEW APPLIANCES AND THINGS MEDICAL.

### PIONEER MILK SUGAR.

(THE PIONEER MILK SUGAR COMPANY, 24 MINORIES, E.C.)

LACTOSE or milk-sugar is a disacharide which, up to the present has only been found in the animal kingdom. It occurs in the milk of all mammals to the extent of from three to five per cent., and its importance in the nutrition of the infant cannot be over-estimated. It differs from the other disacharides: namely, maltose and cane-sugar, in containing after inversion less of a fermentable moiety, it being split up into dextrose and galactose. If we compare from the chemical standpoint human with cow's milk, we find that the former contains roughly about half the proteid and mineral constituents of the latter. From this it follows that if we wish to use cow's milk as a substitute for human milk we must dilute the former with an equal volume of water if we wish them to correspond with regard to their proteid content. Since, however, the lactose and fat percentages in both milks are approximately the same, the simple dilution of cow's milk, without the subsequent addition of lactose and cream, could never even chemically supply us with a substitute for human milk. Under these circumstances it will be obvious how important it is to have at our command a pure milk-sugar. The preparation before us fulfils these conditions, and in our opinion is to be recommended.

